# Effect of abdominal compression on target movement and extension of the external boundary of peripheral lung tumours treated with stereotactic radiotherapy based on four-dimensional computed tomography

**DOI:** 10.1186/s13014-021-01889-0

**Published:** 2021-09-07

**Authors:** Yuanjun Qi, Jianbin Li, Yingjie Zhang, Qian Shao, Xijun Liu, Fengxiang Li, Jinzhi Wang, Zhenxiang Li, Wei Wang

**Affiliations:** 1 Shandong First Medical University and Shandong Academy of Medical Sciences and Now Studies at Shandong Cancer Hospital and Institute , Shandong First Medical University and Shandong Academy of Medical Sciences, Jinan, 250117 China; 2 Department of Radiation Oncology, Shandong Cancer Hospital and Institute, Shandong First Medical University and Shandong Academy of Medical Sciences, 440 Jiyan Road, Jinan, 250117 Shandong Province China

**Keywords:** Peripheral pulmonary tumour, Four-dimensional computed tomography, Stereotactic body radiation therapy, Abdominal compression, Cone beam computed tomography

## Abstract

**Background:**

This study aimed to investigate the effect of abdominal compression on tumour motion and target volume and to determine suitable planning target volume (PTV) margins for patients treated with lung stereotactic body radiotherapy (SBRT) based on four-dimensional computed tomography (4DCT).

**Methods:**

Twenty-three patients diagnosed to have a peripheral pulmonary tumour were selected and divided into an all lesions group (group A), an upper middle lobe lesions group (group B), and a lower lobe lesions group (group C). Two 4DCT scans were performed in each patient, one with and one without abdominal compression. Cone beam computed tomography (CBCT) was performed before starting treatment. The gross target volumes (GTVs) were delineated and internal gross target volumes (IGTVs) were defined. IGTVs were generated using two methods: (1) the maximum intensity projections (MIPs) based on the 4DCT were reconstructed to form a single volume and defined as the IGTVMIP and (2) GTVs from all 10 phases were combined to form a single volume and defined as the IGTV10. A 5-mm, 4-mm, and 3-mm margin was added in all directions on the IGTVMIP and the volume was constructed as PTVMIP_5mm_, PTVMIP_4mm_, and PTVMIP_3mm_.

**Results:**

There was no significant difference in the amplitude of tumour motion in the left–right, anterior–posterior, or superior-inferior direction according to whether or not abdominal compression was applied (group A, *p* = 0.43, 0.27, and 0.29, respectively; group B, *p* = 0.46, 0.15, and 0.45; group C, *p* = 0.79, 0.86, and 0.37; Wilcoxon test). However, the median IGTVMIP without abdominal compression was 33.67% higher than that with compression (*p* = 0.00), and the median IGTV10 without compression was 16.08% higher than that with compression (*p* = 0.00). The median proportion of the degree of inclusion of the IGTVCBCT in PTVMIP_5mm_, PTVMIP_4mm_, and PTVMIP_3mm_ ≥ 95% was 100%, 100%, and 83.33%, respectively.

**Conclusions:**

Abdominal compression was useful for reducing the size of the IGTVMIP and IGTV10 and for decreasing the PTV margins based on 4DCT. In IGTVMIP with abdominal compression, adding a 4-mm margin to account for respiration is feasible in SBRT based on 4DCT.

## Background

Lung cancer has high incidence and mortality rates in both men and women worldwide [1–3], with a 5-year overall survival rate of 68.5–83% in patients with T1N0M0 disease and 53.8–65% in those with T2N0M0 disease [4]. Therefore, there is a good deal of clinical interest in improving early detection and treatment rates [5–7]. Surgical resection is the gold standard treatment for stage T1N0M0 and T2N0M0 non-small cell lung cancer. However, some of these patients have severe cardiovascular disease and poor lung function, and the National Comprehensive Cancer Network guidelines recommend stereotactic body radiotherapy (SBRT) as a standard treatment for those with good local control. SBRT is also suitable for patients with oligometastatic lung tumours [8–12].

Compared with conventional fractionated radiotherapy, SBRT can deliver higher radiation doses in fewer fractions focused on small targets with better local control and survival rates. Furthermore, it protects lung function and shortens the duration of radiotherapy [8, 11–13]. However, the ability of SBRT to provide precise radiotherapy is limited by positional uncertainty in that the position of the targets can be affected by several factors, one of which is respiratory motion [14, 15]. SBRT delivers high radiation doses in each fraction; therefore, technological advances are needed in both imaging and management of respiratory motion to reduce the target margin and limit the risk of complications caused by radiotherapy, especially when the tumour excursion is large [12]. In clinical practice, various manoeuvres can be used to manage motion, included active breath-hold, deep inhalation breath-holding (DIBH), tracking, and abdominal compression.

Active breath-hold is an effective way of reducing the necessary margin due to respiratory motion [16]. Using this procedure, the system is activated when the patient reaches the specified lung volume and stage of the breathing cycle, and the valve is inflated to hold the patient’s breath [14]. DIBH reduces motion in two ways, namely, by deep inspiration, which reduces lung density, and by breath-holding, which prevents movement of the tumour by immobilisation [17]. Patients require respiratory training before receiving this treatment; therefore, it is a hypothesis that poor control of motion using the active breath-hold and DIBH methods could be attributed to poor compliance and respiratory function. Tracking the motion of gold fiducial markers implanted near the tumour is an obvious way of evaluating tumour motion. Using this method, gold spheres or electromagnetic beacons with a diameter of 2 mm are implanted in or near the tumour. The position of each fiducial is tracked in three dimensions several times per second using an imaging system with automatic detection software. The linear accelerator delivers radiation when each fiducial is within an acceptable range of the desired simulation position [14]. However, implantation of fiducials is an invasive procedure for the patient, and assessment of the range of tumour motion is labour-intensive for the clinician [15]. In contrast, the abdominal compression technique requires no respiratory training or open surgery. It simply entails use of a compression plate/belt worn on the abdomen, which adjusts the pressure to a level that is comfortable for the patient and provides an easy-to-use method for complex SBRT setups. Although there have been many studies of the ability of abdominal compression to reduce tumour motion and decrease the target volume for upper abdominal tumours [18–23], several authors have mentioned that abdominal compression sometimes causes interfractional variations and marked respiratory motion.

The aim of this study was to investigate the ability of abdominal compression to reduce tumour motion and the target volume in patients treated with lung SBRT based on four-dimensional computed tomography (4DCT) and to determine the repeatability and conformability of target volumes during SBRT.

## Methods

### Patient characteristics

Twenty-three patients with 32 lesions who received SBRT between August 2018 and March 2020 were prospectively selected in the study if they had the following: a diagnosis of peripheral primary non-small cell lung cancer staged as T1N0M0; (b) a diagnosis of peripheral oligometastatic pulmonary disease that was controlled after initial systemic therapy and a lesion diameter of ≤ 4 cm; (c) an inoperable tumour that was strongly recommended for radiation therapy by a multidisciplinary team; (d) performance status 0–1 and able to breathe freely with abdominal compression; and no thoracic radiotherapy before treatment. Written informed consent was obtained in all cases. The patients were divided into an all lesions group (group A), an upper middle lobe lesions group (group B), and a lower lobe lesions group (group C). The patient and tumour characteristics are listed in Table 1.

**Table 1:** Clinical data of 23 patients (32 lesions) with lung cancer

Characteristic	Number	Proportion of lesions (%)
*Sex*		
Male	15	–
Female	8	–
*Age, years*		
≤ 50	2	–
50–70	16	–
≥ 70	5	–
*Location*		
UL	13^a^ (16 lesions)	50.0
ML	4^a^ (4 lesions)	12.5
LL	10^a^ (12 lesions)	37.5
*Histology*		
SCC	5^a^ (5 lesions)	15.6
Adeno	10^a^ (10 lesions)	31.2
LM	6^a^ (11 lesions)	34.4
Unknown	6 ^a^(6 lesions)	18.8
*Performance score*		
0	21 (30 lesions)	93.8
1	2 (2 lesions)	6.2
*CT performance*		
Solid density lesion	19^a^ (26 lesions)	81.3
Mixed density lesion	6^a^ (6 lesions)	18.7
*Tumor diameter (cm)*		
≤ 2	12^a^ (16 lesions)	50.0
2–3	9^a^ (10 lesions)	31.3
≥ 3	6^a^ (6 lesions)	18.7

*UL* upper lobe, *ML* middle lobe, *LL* lower lobe, *SCC* squamous cell carcinoma, *Adeno* adenocarcinoma, *LM* lung metastases

^a^The total number of patients were not 23 as there were patients who had different site lesions

### CT simulation and image acquisition

During computed tomography (CT) simulation, all patients were immobilised using an abdominal compression system (Body Pro-Lok™; CIVCO Radiotherapy, Orange City, IA, USA) in the horizontal supine position. The respiratory plate places pressure at the level of the diaphragm to assist in restricting respiratory movement. The degree of pressure provided by the plate was as high as the patient could tolerate. Each patient underwent one helical three-dimensional CT (3DCT) and two 4DCT scanning sessions in a Big Bore simulated CT system (Philips Medical Systems, Bensalem, PA, USA). The 3DCT was performed with free-breathing. The first 4DCT was performed with abdominal compression and the second without abdominal compression when free-breathing was restored after removal of the respiratory plate. The patient was asked to breathe freely and rhythmically during both the 3DCT and 4DCT scanning procedures. During acquisition of the 4DCT data, the patient's abdominal surface was tracked using the Sentinel™ system (C-Rad, Uppsala, Sweden) and used as a surrogate for respiratory motion. The respiratory cycle was divided into 10 phases based on classical phase-binning, and reconstructed CT images were defined as CT0, CT10, CT20 through to CT90, where CT0 represents the dataset from end-inhalation and CT50 represents the dataset from end-exhalation during one respiratory cycle. The maximum intensity projection (MIP) was reconstructed from all 10 4DCT phases, referred to as MIP_com_ and MIP_non-com_ for images obtained with abdominal compression and free-breathing, respectively. All patients were treated using a linear accelerator (VitalBeam™, Varian Medical Systems, Palo Alto, CA, USA). Before treatment, cone beam CT (CBCT) of the chest was performed based on the position described earlier for the CT simulation. CT and CBCT images were acquired from the cricothyroid membrane to 5 mm beneath the diaphragm with a thickness of 3 mm. All images were transferred to the treatment planning system (MIM software version 6.7.6; MIM Software Inc., Cleveland, OH, USA).

### Target definition

**Fig. 1:**
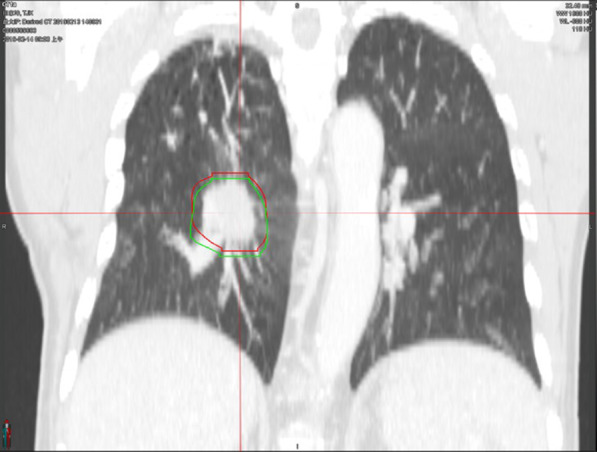
Gross tumor volume (GTV) delineation with and without abdominal compression (AC). Red delineation was for GTV with AC, green delineation was for GTV without AC

To eliminate interobserver variation, the target volumes were delineated on the CT images by the same physician using the MIM software. The gross target volumes (GTVs) were delineated on images from all phases of 4DCT with or without compression (GTV4DCT_com_ and GTV4DCT_non-com_, respectively) in the same window width of 1600 HU with a window level of − 600 HU. [24] The GTVs delineated with and without abdominal compression are shown in Fig. 1. The internal gross target volume (IGTV) was generated using two methods: (1) MIPs based on the 4DCT were combined to form a single volume (IGTVMIP) and (2) GTVs from all 10 phases were combined to form a single volume (IGTV10).

### Tumour motion and volume

The excursion of the GTVs was compared. The coordinates of the centre-of-mass of the target volume calculated using GTV4DCT_com_ and GTV4DCT_non-com_ were recorded to analyse the motion amplitudes of GTVs in different modes using the following formula:

$$\Delta = \sqrt {\Delta X^{2} + \Delta Y^{2} + \Delta Z^{2} } .$$

where ΔX, ΔY, and ΔZ are defined as the movement in the left–right (LR), anterior–posterior (AP), and superior-inferior (SI) directions, respectively. The IGTVMIP and IGTV10 values with and without compression were compared.

### Target consistency

The PTVMIP_5mm_, PTVMIP_4mm_, and PTVMIP_3mm_ were created by addition of a 5-mm, 4-mm, and 3-mm margin, respectively, to the IGTVMIP_com_ in the LR, AP, and SI directions. The degree of inclusion (DI) between IGTVCBCT and PTVMIP_com_ was used to define the repeatability and conformability of PTVMIP_com_ relative to IGTVCBCT.

The formula used to calculate DI was as follows:

$${\text{DI }} = \, \left( {{\text{X }} \cap {\text{ Y}}} \right){\text{/X}}.$$

X ∩ Y is defined as the overlap between the IGTV and PTV values (MIM software, 6.7.6).

Assuming volume X to be the reference for the standard volume, if the treatment planning was based on volume Y, 1-DI (X in Y) of volume X would be irradiated unnecessarily and 1-DI (X in Y) of volume Y would miss irradiation [24]. And in this study, X = IGTVCBCT and Y = PTVMIP_com_.

### Statistical analysis

The target motion and volume data were not normally distributed. Therefore, the Wilcoxon test was used to compare the target motion and volume. The statistical analysis was performed using SPSS for Windows version 23.0 (IBM Corp., Armonk, NY, USA). A *p* value < 0.05 was considered statistically significant.

## Results

Table 2 shows the GTV excursion at different sites in the lungs. When compared with non-compression, the median GTV excursion with compression increased by 6.67% in the LR direction but decreased by 6.45% in the AP direction and by 29.41% in the SI direction in group A, increased by 71.43% in the LR direction and reduced by 7.69% and 40.00% in the AP and SI directions, respectively, in group B, and did not change in the LR direction but increased by 5.56% in the AP direction and decreased by 54.69% in the SI direction in group C. There was no statistically significant between-group difference in GTV excursion according to whether or not compression was applied. The median 3D GTV motion with compression and non-compression was 3.15 mm (range 0.05–11.10) and 4.56 mm (range 0.41–12.92), respectively, in group A, 3.02 mm (range 0.05–7.38) and 2.92 mm (range 0.41–12.92), respectively, in group B, and 5.40 mm (range 2.12–11.10) and 6.65 mm (range 4.01–11.28), respectively, in group C; there was no significant difference between compression and non-compression in the three groups (*Z* = −1.16, − 1.17, − 0.65, respectively; *p* = 0.25, and 0.24, 0.52). In group B, comparing compression with non-compression, there was an obvious increase for 7 (35%), 6 (30%),8 (40%), and 7 (35%) of the 20 targets in the LR, AP, and SI directions and the 3D vector, respectively; a clear reduction for 11 (55%), 11 (55%), 10 (50%), and 12 (60%) of the 20 targets in the LR, AP, and SI directions and the 3D motion, respectively; and no change in the target motion amplitude of the 20 targets in the LR, AP, or SI direction or in the 3D motion for the 2 (10%), 3(15%), 2 (10%), and 1 (5%) remaining targets. In group C, comparing abdominal compression with non-compression, there was an obvious increase for 2 (17%), 5 (42%), 4 (33%), and 5 (42%) of the 12 targets in the LR, AP, and SI directions and in the 3D motion, respectively; an obvious reduction for 3 (25%), 4 (33%), 5 (42%), and 4 (33%) of the 12 targets in the LR, AP, and SI directions, and in the 3D motion, respectively; and no change in the target motion amplitude for the 7 (58%), 3 (25%), 3 (25%), and 3 (25%) remaining targets in the LR, AP, or SI direction or in the 3D motion.

**Table 2:** Median and range of centroid shifts with and without compression in the LR, AP, and CC directions for group A, group B, and group C (mm)

Group	LR direction		AP direction		SI direction	
	Com	Non-com	Com	Non-com	Com	Non-com
*Group A*						
Median	0.80	0.75	1.45	1.55	2.40	3.40
Range	0.10–4.00	0.20–4.60	0.10–4.40	0.20–4.50	0.10–10.00	0.00–11.70
*p*	0.43		0.27		0.29	
*Group B*						
Median	1.20	0.70	1.20	1.30	1.50	2.50
Range	0.10–2.00	0.20–4.60	0.10–3.20	0.20–4.50	0.10–6.50	0.00–11.70
*p*	0.46		0.15		0.45	
*Group C*						
Median	1.00	1.00	1.90	1.80	2.90	6.40
Range	0.20–4.00	0.40–3.40	0.30–4.40	0.60–2.80	0.90–10.00	2.80–11.00
*p*	0.79		0.86		0.370.37	

*LR* left–right, *AP* anterior–posterior, *CC* cranial–caudal, *com* compression, *non-com* non-compression, *Group A* all lesions group, *Group B* upper-middle-lobe lesions group, *Group C* lower-lobe lesions group

The median IGTVMIP_com_ and IGTVMIP_non-com_ volumes in group A were 4.01 cm^3^ (range 0.39–34.84) and 5.36 cm^3^ (range 0.41–41.90), respectively; the difference was statistically significant (*Z* = −3.45, *p* = 0.00; Wilcoxon test). The median IGTV10_com_ and IGTV10_non-com_ volumes in group B were 6.59 cm^3^ (range 0.45–36.89) and 7.65 cm^3^ (range 0.43–46.46), respectively, and also significantly different (*Z* = −3.14, *p* = 0.00; Wilcoxon test).

When a 5-mm margin was added to the IGTVMIP_com_ to form the PTVMIP_com_, the median DI of the IGTVCBCT in the PTVMIP_5mm_ was 100%. When 4-mm and 3-mm margins were added, the median proportions of the DI in IGTVCBCT in PTVMIP_4mm_ and PTVMIP_3mm_ ≥ 95% were 100% and 83.33%, respectively.

## Discussion

SBRT is a highly conformal and hypo-fractionated type of radiotherapy used to treat lung cancer with high‐dose radiation that can be focally administered and with exquisite dose fall-off [8, 25]. The main limiting factor for lung SBRT is respiration, which can lead to larger PTVs during treatment and an excessive risk of radiation‐induced complications. The tumour tissue and normal tissue treated with SBRT would receive different radiation doses under different breathing conditions [26]; therefore, management of respiratory movement could increase the benefit of radiotherapy for tumours in the lungs and other organs that are markedly affected by respiratory motion. Accurate measurement of tumour excursion is an essential component of management of respiratory movement in patients with lung tumours. In these patients, the effect of amplitude of respiratory movement depends on many factors, including the location of the tumour. Different measurement methods could also lead to different results. Therefore, there is considerable variation in the findings reported in the literature [27, 28]. Takao et al. [27] reported that the amplitude of baseline shift/drift of lung tumours in the craniocaudal direction was 1.65 mm using a real-time tumour-tracking radiation therapy system in 68 patients treated using SBRT with free-breathing. In a study that included 20 lesions, Negoro et al. [28] found that the average tumour excursion was 7.7 mm in the craniocaudal direction under free respiration using X-ray fluoroscopy simulation. In the present study, we calculated that the median excursion was 3.4 mm (2.5 mm for the upper middle lobe and 6.4 mm for the lower lobe) in the SI direction under free respiration using 4DCT.

Management of respiratory movement during lung SBRT aims to eliminate or minimise the target excursion caused by respiratory motion and to decrease the IGTV. Although abdominal compression has been considered to be an effective method for reducing respiratory movement, its effects on tumour excursion had not been consistent in the published reports [29–32]. Negoro et al. [28] reported that abdominal compression achieved a significant reduction in movement of tumours in the lower lobe from a range of 8–20 to 2–11 mm using fluoroscopic X-ray simulation. Bouihol et al. [29] found a reduction in tumour excursion in 82% of cases, with a reduction of 3.5 mm in the lower lobe and 0.8 mm in the upper middle lobe; however, case analysis indicated that the tumour excursion increased under abdominal compression in five cases using 4DCT. Mampuya et al. [30] reported that compression could reduce tumour excursion in the craniocaudal direction from 19.9 ± 7.3 to 12.4 ± 5.8 mm for tumours with excursion > 8 mm when measured using CBCT. Javadi et al. [31] reported tumour excursion of 6.1 mm with compression and 6.0 mm without compression in patients treated with lung SBRT, which suggested that abdominal compression could not reduce tumour excursion. Their finding is in accordance with that in a study by Rasheed et al. [32] who reported that tumour excursion was reduced in three patients, increased in five, and remained the same in nine. Overall, in that study, abdominal compression did not have any significant effect in reducing tumour excursion. The authors suggested that the effect of compression may be patient-specific and that the lobe in which the tumour was located did not predetermine the efficacy of compression. In our study, there was no statistically significant difference in the effect of compression on tumour excursion according to whether the tumour was in an upper middle lobe or a lower lobe. However, in numerical terms, the excursion in the SI direction decreased with abdominal compression regardless of tumour location, and this change in the tumour excursion pattern may explain the difference in IGTV.

Compared with non-compression, we found that abdominal compression could significantly reduce the IGTV, specifically, IGTVMIP_com_ < IGTVMIP_non-com_ (median reduction of 1.35 cm^3^) and IGTV10_com_ < IGTV10_non-com_ (median reduction of 1.06 cm^3^). Bouihol et al. [29] showed that abdominal compression could reduce the internal target volume (ITV) of tumours by approximately 1.3 cm^3^ for lung SBRT, which is consistent with our present findings. The reduction in IGTV (ITV) might reduce the incidence of radiation-induced lung injury [30]; therefore, abdominal compression may be beneficial for lung SBRT.

CBCT is an effective way of confirming the effect of respiratory management techniques on SBRT. At present, 4DCT and CBCT are widely used in SBRT for lung tumours. Calculating the DI of IGTVCBCT_com_ in PTVMIP_com_ could confirm the repeatability and consistency of the target during radiotherapy [33–35]. The American Association of Physicists in Medicine proposed differential margins for the PTV in all three dimensions during lung tumour SBRT [15]; undoubtedly, the excessive target volume increased the amount of unnecessary irradiation delivered to the surrounding normal lung tissue. [36] Therefore, lung tumour SBRT is usually expanded by 5 mm from the IGTV to construct the PTV. The results of our study indicate that an IGTVMIP_com_ with a 4-mm margin to account for respiration is reasonable for patients treated with SBRT under abdominal compression. Of course, the reduction in the PTV expansion boundary was based on positioning error and online position correction based on CBCT; therefore, automatic registration, positioning error correction, and manual registration based on soft tissue landmarks must be carried out before each CBCT-based treatment. If based only on bony automatic registration, abdominal compression is not recommended for lung SBRT because it can lead to uncorrected interfractional motion [29, 37], adversely affect local control of the tumour, and reduce patient survival [20].

We found that when patients with peripheral lung tumours were treated with SBRT, abdominal compression did not significantly change the tumour excursion in any of the three dimensions or the overall motion vector of the lung tumour and that the respiratory pattern changed in all lobe locations. Patients tend to breathe with the upper thoracic region when the lower region is immobilised; therefore, when abdominal breathing is suppressed, chest breathing is enhanced, which leads to changes in the excursion of tumours in different directions of the lung [29, 31]. Our results are more meaningful in terms of the efficacy of compression on target volumes. Abdominal compression reduced the IGTV, increased the DI of IGTVCBCT_com_ in PTVMIP_com_, and reduced the PTV margins based on 4DCT.

The main limitation of this study was its small sample size, especially in the group of patients with tumours in the lower lobes, which led to smaller tumour excursions. Further studies with larger case numbers are needed to confirm our findings.

## Conclusions

Use of abdominal compression did not decrease the motion of peripheral lung tumours in this study. When compared with non-compression, the GTV motion was not significantly decreased in any of the three directions measured or in the 3D vector regardless of the site of the tumour in the lung. While there was a change in the respiratory pattern with compression, it was related to the lung lobes in that the excursions increased in the LR direction and decreased in the AP and SI directions for the upper middle lobes but did not change in the LR direction, increased in the AP direction, and decreased in the SI direction for the lower lobes. A substantially smaller IGTV was seen for peripheral pulmonary tumours, and compression could increase the DI of IGTVCBCT_com_ in PTVMIP_com_ to minimise the external extension boundary of peripheral pulmonary tumours treated with SBRT based on 4DCT.

## Data Availability

All data generated or analyzed during this study are included in this published article. The authors in this article insure the availability of supporting data.

## Abbreviations

4DCT: Four-dimensional computed tomography
AP: Anterior–posterior
CBCT: Cone beam computed tomography
DI: Degree of inclusion
DIBH: Deep inhalation breath-holding
GTV: Gross target volume
IGTV: Internal gross target volume
ITV: The internal target volume
LR: Left–right
MIP: Maximum intensity projection
PTV: Planning target volume
SBRT: Stereotactic body radiotherapy
SI: Superior–inferior
